# Tissue-engineered repair material for pelvic floor dysfunction

**DOI:** 10.3389/fbioe.2022.968482

**Published:** 2022-09-06

**Authors:** Meina Lin, Yongping Lu, Jing Chen

**Affiliations:** ^1^ NHC Key Laboratory of Reproductive Health and Medical Genetics (China Medical University) and Liaoning Key Laboratory of Reproductive Health, Liaoning Research Institute of Family Planning (The Affiliated Reproductive Hospital of China Medical University), Shenyang, China; ^2^ Department of Obstetrics and Gynecology, Shengjing Hospital of China Medical University, Shenyang, China

**Keywords:** pelvic floor dysfunction, tissue-engineered repair material, mesenchymal stem cells, scaffolds, pelvic organ prolapses, stress urine incontinence

## Abstract

Pelvic floor dysfunction (PFD) is a highly prevalent urogynecology disorder affecting many women worldwide, with symptoms including pelvic organ prolapse (POP), stress urinary incontinence (SUI), fecal incontinence, and overactive bladder syndrome (OAB). At present, the clinical treatments of PFD are still conservative and symptom-based, including non-surgical treatment and surgery. Surgical repair is an effective and durable treatment for PFD, and synthetic and biological materials can be used to enforce or reinforce the diseased tissue. However, synthetic materials such as polypropylene patches caused a series of complications such as mesh erosion, exposure, pain, and inflammation. The poor mechanical properties and high degradation speed of the biomaterial meshes resulted in poor anatomical reduction effect and limitation to clinical application. Therefore, the current treatment options are suboptimal. Recently, tissue-engineered repair material (TERM) has been applied to repair PFD and could markedly improve the prognosis of POP and SUI repair surgery in animal models. We review the directions and progression of TERM in POP and SUI repair. Adipose-derived stem cells (ADSCs) and endometrial mesenchymal stem cells (eMSCs) appear to be suitable cell types for scaffold seeding and clinical implantation. The multidisciplinary therapy approach to tissue engineering is a promising direction for tissue repair. More and longer follow-up studies are needed before determining cell types and materials for PFD repair.

## Graphical Abstract

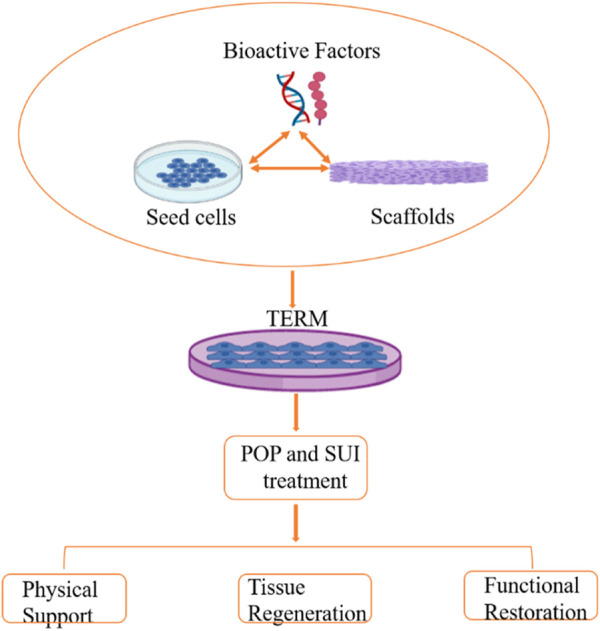

## Introduction

Pelvic floor dysfunction (PFD) is a highly prevalent urogynecology disorder, affecting about 30%∼50% of middle-aged and elderly women. The main manifestations of PFD are pelvic organ prolapse (POP), stress urinary incontinence (SUI), fecal incontinence, and overactive bladder syndrome (OAB). POP and SUI mostly occur in parous women and are caused by childbirth-associated pelvic floor injury. The prevalence of PFD varies in different geographical regions. It is estimated that one in three and one in nine women are affected by SUI and POP, respectively, and POP and SUI may coexist in up to 80% of women with prolapse (Farmer et al., 2020). Studies have predicted that the number of women with at least one PFD will increase by 55.8% from 2010 to 2050 (Wu et al., 2009).

At present, the clinical treatments of PFD are still conservative and symptom-based, including non-surgical treatment and surgery. Non-surgical treatment mainly consists of a manual approach, stimulation, or relaxation technique (Quaghebeur et al., 2021). Surgery was considered to be the most effective and durable treatment, which includes autologous tissue repair and synthetic mesh implantation. In general, autologous tissue repair showed good integration within host tissues, a minimal to moderate inflammatory response, and a moderate degree of collagen production and underwent a degree of remodeling over the long term, but a high recurrence rate (Gigliobianco et al., 2015; Lo et al., 2015). Polypropylene patches are the most used synthetic meshes in implantation surgery, which offer several advantages including lack of transmission of infectious diseases, ease of availability, sustainable tensile strength, and good mechanical properties. However, its nondegradable nature and poor histocompatibility caused a series of problems such as mesh erosion, exposure, pain, infection, pronounced inflammation, massive cell infiltration, and collagen production, and these led to the withdrawal of transvaginal polypropylene mesh from the market and its banning in some countries (Elmer et al., 2009; Gigliobianco et al., 2015). Biomaterial meshes have been used recently for their good histocompatibility, but the poor mechanical properties and high degradation speed resulted in poor anatomical reduction effect and limitation to clinical application. Therefore, the current treatment options are suboptimal, and alternative methods are needed to promote the repair of PFD.

The defects of the pelvic floor support, which are due to the interaction between the muscles and connective tissues within the pelvis, are the main pathophysiology of PFD. They are mainly manifested as altered elastin/collagen metabolism and connective tissue abnormalities (Jin et al., 2016a), such as (1) the interruption in elastin homeostasis caused by abnormal degradation/synthesis of elastin and (2) the changes in the total content and the ratio of subtypes of collagen and the deficient crosslinking of collagens. Therefore, the goal of PFD therapy is to support weakened tissue of the pelvic floor and promote tissue remodeling, thus achieving the effect of improving pelvic floor functions.

**FIGURE 1:**
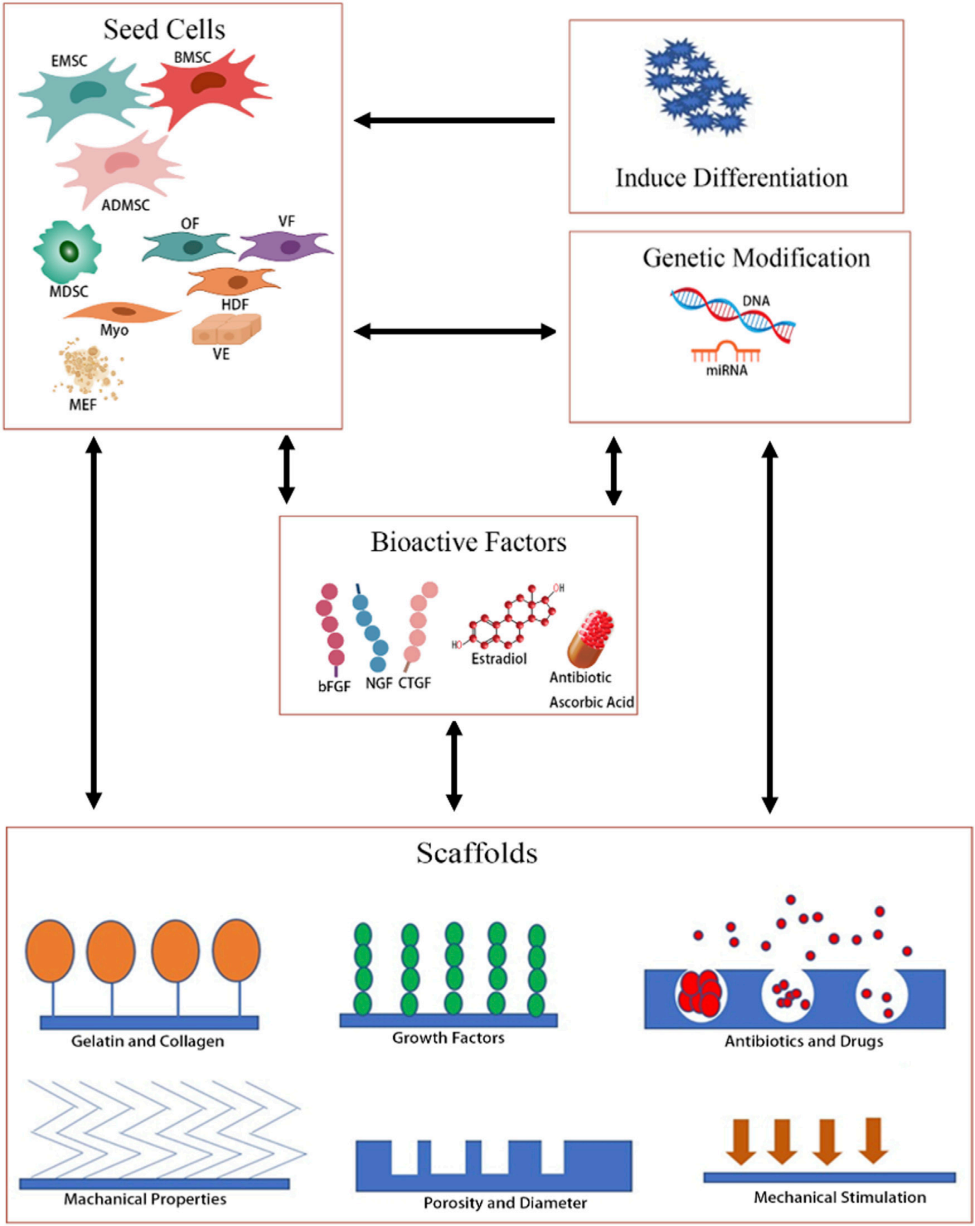
Overview of TERMs of different strategies for PFD. OF, oral fibroblast; VF, vaginal fibroblast; HDF, human dermal fibroblast; VE, vaginal epithelial; Myo, myoblasts; MFF, muscle fiber fragment.

Tissue-engineered repair material (TERM) is a multidisciplinary therapy approach that seeks to use a combination of cells, materials, and sometimes additional genes, drugs, or growth factors, to regenerate diseased tissues. The purpose of the cell component, gene, and growth factor is to accelerate repair and promote regeneration of damaged or lost tissue, while the material provides physical support and niches to deliver cells, drugs, and growth factors to the tissue, which, in turn, provides a platform to control the local pharmacokinetics of growth factors and the proliferation and differentiation of transplanted stem cells (Mangir et al., 2019; Zhao et al., 2021). Using TERM for PFD treatment has obvious advantages: first, the scaffolds can support weakened tissue and will not cause so many complications for its degradable nature and good histocompatibility; second, the seeding cells will drive tissue remodeling, ultimately providing a permanent repair; third, the bioactive factors, such as growth factors, drugs, and estrogen can speed up repair and improve the microenvironment. So, TERM is a promising treatment for PFD. However, the choice of cells and scaffolds is crucial to the success of TERM. Therefore, different scaffolds and cells have been studied to create a TERM for the treatment of PFD. So, we reviewed the research results of TERM applied in PFD treatment and drew the structure diagram of TERM (Figure 1).

### Seed cells

Seed cells are the premise of tissue engineering; they could be obtained from a wide range of sources, including autologous, allogeneic, and heterologous tissues. Stem cells and differentiated cells have been reported as the seed cells for pelvic floor repair. Mesenchymal stem cells (MSCs) are the most widely used stem cells, including adipose-derived stem cells (ADSCs), bone marrow-derived mesenchymal stem cells (BMSCs), endometrial mesenchymal stem cells (eMSCs), and muscle-derived stem cells (MDSCs). The differentiated cells include fibroblasts, myoblasts, and smooth muscle cells. Among them, ADSCs and eMSCs are the most promising cells for experimental research and clinical treatment of PFD (Table 1).

**TABLE 1:** Advantages and disadvantages of different seed cells and scaffolds.

Seed cell	Advantage	Disadvantage
ADSCs	(1) Adipose tissue is readily available in large quantities during single liposuction and without general anesthesia	N/A
	(2) Easy to culture and expand *in vitro*, self-renewal and multilineage differentiation ability, and proliferative efficiency	
	(3) Low donor morbidity and surgical interference	
	(4) Good compatibility with biological materials	
eMSCs	(1) Excellent regenerative capacity in the endometrial lining	N/A
	(2) Easily obtained from menstrual blood or endometrial biopsies, even from the post-menopausal uterus	
	(3) Procurement method is minimally invasive, with minimum pain and morbidity, without anesthesia	
	(4) Easy to culture and expand *in vitro*, self-renewal and multilineage differentiation ability, and high proliferative capacity *in vitro*	
	(5) Can be purified using a unique marker SUSD2	
	(6) A83-01 can maintain clonogenic SUSD2^+^eMSCs and prevent spontaneous fibroblast differentiation	
	(7) EMSCs could reduce the foreign body reaction to the degradable mesh	
BMSC	(1) Easy to culture and expand *in vitro*, self-renewal and multilineage differentiation ability, and high proliferative capacity *in vitro*	(1) The procurement method is invasive, with pain and need for general anesthesia
	(2) BMSCs are used in the repair of various diseases and injuries with good efficacy	(2) Relatively scarce number and low cell yields, especially cannot be easily expanded in the middle-aged people
MDSC	(1) Could form myotubes	Acquisition requires surgical anesthesia and invasive operation, resulting in pain and low cell yield
	(2) Could improve the function of urination in rats with intrinsic sphincter deficiency and increase the expression of myosin and α-SMA.	
Fibroblasts	(1) Similar to the nature of the cells in the damaged tissue, it can repair the damaged tissue	Acquisition requires surgical anesthesia and invasive operation, resulting in pain and low cell yield
Scaffold		
PLA	(1) Mimics the architecture of native fascia tissues and integrates well into native tissues	(1) Too brittle and degrades very slowly *in vivo*
	(2) Produces better extracellular matrix components	(2) Acidity and high crystallinity of its byproduct degradation often triggered inflammatory reactions
	(3) With good cell infiltration, neovascularization, and macrophage type 2 response	
PCL	(1) Good thermal stability, good biocompatibility, and low immunogenicity	Hydrophobicity of PCL impedes cell adhesion and limits the degradation rate
	(2) Easy to process and surface modifications	
PLGA	(1) Good biocompatibility and controllable biodegradability	Limited mechanical properties
	(2) Can be combined with a variety of materials	

ADSCs are considered beneficial for PFD treatment by *in vitro* and *in vivo* studies because ADSCs can be obtained in large quantities during single liposuction and without general anesthesia and are easy to culture and expand *in vitro* (Chen H. et al., 2021a; Wang et al., 2021). ADSCs not only exhibit self-renewal and multilineage differentiation, but they also have confirmed proliferative efficiency and low donor morbidity (Sterodimas et al., 2010). In addition, there is a relatively low occurrence of surgical interference in collecting them as compared with other derived stem cells. Some studies suggested that ADSCs have equal or superior therapeutic potential compared to BMSCs. ADSCs were used to treat SUI in many clinical trials by urethral injection showing better results and minimal side effects and complications (Barakat et al., 2020). Roman et al. (2014) showed that hADSCs appear to be suitable cell types to combine with biodegradable scaffolds for the treatment of SUI and POP.

eMSCs are a rare population of perivascular MSCs in the endometrial layer of the uterus and are another ideal source of autologous therapeutic cells for the treatment of POP using novel tissue engineering approaches for the following reasons: (1) eMSCs exhibit excellent regenerative capacity in the endometrial lining following menstruation and high proliferative capacity *in vitro*. (2) They can be easily obtained from menstrual blood or endometrial biopsies, even from the post-menopausal uterus; the procurement method is minimally invasive, without the need for an anesthetic, resulting in minimum pain and morbidity. (3) eMSCs can be purified using a unique marker, SUSD2 (Masuda et al., 2012). (4) A small molecule TGF-β receptor inhibitor (A83-01) can maintain clonogenic SUSD2^+^eMSCs and prevents spontaneous fibroblast differentiation during culture expansion (Gurung et al., 2015). (5) eMSCs could reduce the foreign body reaction to the degradable mesh by modulating inflammatory cytokine secretion and changing the ratio of M1/M2 of macrophages (Darzi et al., 2018).

BMSCs are among the best characterized of all stem cells studied so far, with great differentiation ability and the ability to secrete factors beneficial to tissue repair. BMSCs are used in the repair of various diseases and injuries with good efficacy in many studies. Among them, the treatment of PFD by BMSCs was also studied *in vitro* and *in vivo* and the results showing good repair effects, whether through local cell injection or tissue engineering combined with biological scaffolds (Jin et al., 2016a; Jin et al., 2016b; Zhao et al., 2017; Zhao et al., 2018; Zhao et al., 2021). However, BMSCs carry disadvantages such as painful isolation procedures, the need for general anesthesia, relatively scarce numbers, and low cell yields, and they, especially, cannot be easily expanded in middle-aged people. So these shortcomings limited the clinical use of BMSCs.

MDSCs are isolated from muscle biopsies and differentiated *in vitro* and *in vivo* into skeletal myotubes, osteoblasts, chondrocytes, neural cells, and smooth muscle cells. There are only small studies investigating PFD treatment using MDSCs with or without scaffolds. The clinical study showed that the quality of life in patients with SUI improved significantly 2−4 years after the MDSC injection procedure (Stangel-Wojcikiewicz et al., 2014; Stangel-Wojcikiewicz et al., 2016). *In vitro* studies demonstrated that urethral striated muscle-derived stem/progenitor cells (uMDSCs) could form myotubes when treated with micro-energy acoustic pulses or low-intensity extracorporeal shock waves (Wang B. et al., 2018a; Cui et al., 2019). Animal experiments showed that MDSC injection improved the function of urination in rats with intrinsic sphincter deficiency and increased the expression of myosin and α-smooth muscle actin (α-SMA) (Cui et al., 2018). However, the acquisition of MDSCs requires surgical anesthesia and invasive operation, resulting in pain and low cell yield, which limits its clinical application.

Additionally, some other differentiated cells were also used for repairing damaged tissue due to PFD, such as vaginal fibroblasts, epithelial cells, smooth muscle cells, and human oral fibroblasts. However, the use of these differentiated cells in tissue engineering repair is significantly less than that of MSCs, for MSCs have a particular advantage over differentiated cells.

## Scaffolds

The ideal scaffolds for tissue engineering should meet the following conditions: (1) the scaffolds should be biocompatible, integrate well into the patient’s tissues, and reflect the properties of the tissues into which it is implanted. (2) The property of scaffolds should be stable, with proper porosity and pore size, conducive to cell adhesion, proliferation, no toxicity, and no immunity. (3) The scaffolds should be strong enough to provide structural support and remain relatively elastic to cope with the forces experienced with routine events such as coughing or sneezing and become reversibly stronger at higher strain, similar to the native healthy fascia. (4) Materials proposed for grafts should degrade *in vivo*, provoke an acute inflammatory response, undergo tissue remodeling, allow cell permeability, and exhibit mechanical robustness at the point of implantation (Gigliobianco et al., 2015). PFD is a chronic disorder with no optimal standards for the repair or treatment; an ideal mesh would be desirable for degradable biomaterials to last 6–12 months to ensure sufficient tissue re-organization with desired stiffness. The scaffolds in TERM for PFD were mainly synthetic materials in a large number of *in vitro* and *in vivo* studies, and they are all FDA-approved materials, such as poly-L-lactic acid (PLA), polyglycolide (PGA), poly lactic-co-glycolic acid (PLGA), and polycaprolactone (PCL). Different materials have their own advantages and disadvantages, and they had been reported in the study of PFD treatment by combining with different cells (Table 1).

PLA scaffold mimics the architecture of native fascia tissues, produces better extracellular matrix components for cell attachment and proliferation *in vitro*, and integrates well in native tissue with good cell infiltration, neovascularization, and macrophage type 2 (M2) response (Roman et al., 2019). However, PLA is too brittle and degrades very slowly *in vivo*, and the acidity and high crystallinity of its degradation byproducts often triggered inflammatory reactions. Relatively few studies were reported on the effect of PLA or PLA-added cells on PFD (Mangir et al., 2016; Roman et al., 2016; Mangir et al., 2019).

PCL is an FDA-approved polyester with excellent thermal stability, good biocompatibility, and low immunogenicity, it is easy to process and susceptible to surface modifications, and it has been widely used as biomaterials for tissue engineering purposes, including pelvic meshes (Siddiqui et al., 2018). However, the hydrophobicity of PCL impedes cell adhesion and limits the degradation rate. So many PCL composites were studied for PFD treatment, such as PCL/PLA, PCL/PGA, PCL/PEG (Ren et al., 2022), PLGA/PCL (Vashaghian et al., 2017; Vashaghian et al., 2019; Chen YP. et al., 2021b), and UPy-PCL(Hympanova et al., 2018), and the results showed that PCL/PEG and PLGA/PCL were better than the others, the degradation rate of PCL/PLA and PCL/PGA were too high, and UPy-PCL had a high failure rate.

PLGA scaffold is approved by the FDA (U.S. Food and Drug Administration, United States)/EMA (European Medicines Agency) and extensively explored. PLGA holds a prominent position in a variety of tissue repairs due to its biocompatibility and controllable biodegradability. PLGA scaffold has been successfully applied in pelvic floor repair (Oe et al., 2022) and bladder replacement alone (Salem et al., 2020) (Rocha et al., 2022) or PLGA-based composites, such as PLGA-NPs (Jin et al., 2016a; Jin et al., 2016b), PLGA/PCL (Qian et al., 2016; Vashaghian et al., 2016), PLGA-MPEG (Jangö et al., 2017), and PLACL/gelatin (Mukherjee et al., 2019). Among them, PLGA/PCL has been studied the most in the treatment of pelvic floor repair.

In addition, other scaffolds are also used for PFD repair, such as PGA, PLTG, PU, and PLCL/Fg (Hou et al., 2014; Wu et al., 2016; Wang et al., 2017; Wang X. et al., 2018b; Masudi and Abdelrahman, 2021). However, the relevant studies are limited. PGA is beneficial to using as sling material for SUI treatment because of its low stiffness.

### Bioactive factors

The appropriate microenvironment for tissue regeneration is another key factor of an ideal tissue engineering scaffold. So some bioactive factors were added to scaffolds to induce stem cell proliferation and differentiation, promote cell–material interaction, enhance mechanical and biological properties, and antisepsis, etc. PFD-related factors include basic fibroblast growth factor (bFGF), nerve growth factor (NGF), connective tissue growth factor (CTGF), estrogen, 17-β-estradiol, ascorbic acid, ascorbate-2-phosphate, and antibiotics. Jin et al. demonstrated that bFGF significantly promoted the production of collagen and elastin from elastin-expressing BMSCs *in vitro* and *in vivo*, and co-injection of PLGA-loaded bFGF NP and elastin-expressing BMSCs into the PFD rats significantly improved the outcome of urodynamic tests (Jin et al., 2016a). *In situ* gel-forming bulking agent, containing NGF and bFGF, provided regeneration of damaged nerves and smooth muscles and enhanced biological function around the urethra (Oh et al., 2015). The solid PCL-CTGF mesh delivering rMSC demonstrated improved biomechanical properties and no complications after implantation in a rat model of a weakened vaginal wall after 8−24 weeks (Hansen et al., 2020). Studies have shown that estrogen released from PLA mesh promotes more ECM production involving collagen I, collagen III, and elastin and doubled new blood vessel formation in the chorioallantoic membrane (Mangir et al., 2019). Shafaat et al. (2018) reported that 17-β-estradiol-releasing PU scaffolds showed increased ultimate tensile strength and produced more ECM and blood vessels. However, the controversial therapeutic effect of estrogen was also reported (Wu et al., 2020). Ascorbic acid and ascorbate-2-phosphate in the PLA scaffold could increase its hydrophilicity and strength and promote the production of collagen from human dermal fibroblasts (Mangir et al., 2016). Ren et al. (2022) coated the PCL/PEG composite meshes with azithromycin and elicited the antibacterial properties of the meshes; the results showed that it is effective in suppressing the growth of *S. aureus* bacteria and will be supporting cell attachment and proliferation.

### The cross-talk between scaffolds and cells

Cell therapy using MSCs and surgery with synthetic polymer scaffolds alone could somewhat improve the PFD symptoms, and cell-based tissue engineering has even more dramatic therapeutic effects. So, how and why therapeutic cells together with scaffolds enhance therapeutic outcomes is the key factor. From a clinical perspective, the cross-talk between scaffolds and cells may be imperative for enhancing the therapeutic effect of PFD (Mukherjee et al., 2019).

The cell–biomaterial interactions between eMSCs and nanofiber meshes of PLACL/gelatin *in vitro* and *in vivo* were evaluated by Mukherjee et al. (2019), and the results showed: (1) mesh properties influence eMSC size, proliferation rate, adhesion, migration, structure, and expression of F-actin and vinculin protein *in vitro*. PLACL/gelatin nanofiber meshes provide suitable scaffolding where cells can interact with the mesh, as well as with each other and the host cells. (2) PLACL/gelatin significantly enhanced eMSC retention and infiltration properties *in vivo*. Many clinical trials of cell therapies suggested that mere injection of therapeutic cells is not sufficient to cure diseases (Galipeau and Sensebe 2018). One reason is that there are no enough cells in the damaged site, so retention of MSCs in the transplantation site is a key factor for cellular infiltration and tissue integration. (3) eMSCs control ECM formation and degradation of nanofiber meshes *in vivo*, allowing time for tissue strengthening before the scaffold fully disappears. (4) eMSCs influence the macrophage-based foreign body response to nanofiber meshes *in vivo*. Wang X. et al. (2018b) reported that the addition of ADSCs onto both PLA and PLTG scaffolds led to a significant increase in stiffness and maximum stress of the scaffolds. A similar phenomenon was also observed in other experiments using human oral fibroblasts (OFs) and the hADSC effects on PLA scaffold (Roman et al., 2014). Significant temporal changes in tensile properties and clear differences in collagen organization with time between eMSC-seeded and -unseeded scaffolds were observed (Edwards et al., 2015). PLA combined with PU greatly improved the interaction of cells with this material (Hillary et al., 2016).

So, the cross-talk between scaffolds and cells is of great significance in tissue engineering. The scaffolds could provide support for cell growth, promote cell proliferation, and prevent cell migration. At the same time, the cells can affect the property of the biomaterials, such as stiffness, elasticity, and degradation rate, especially for PFD, it would be desirable for degradable biomaterials to last 6–12 months until new tissue of desired stiffness has been regenerated, so regulating the degradation of biomaterials is the key to TERM for PFD (Hillary et al., 2016).

### Tissue-engineered repair material for stress urinary incontinence

SUI is the involuntary urine leakage associated with increased intra-abdominal pressure on the bladder during activities, such as coughing, laughing, sneezing, exercising, impact movements, or squatting (Wallace et al., 2019). The urine leakage of SUI happens in the absence of detrusor contraction. The pathophysiology of SUI is not well known and multifactorial, including pregnancies with subsequent vaginal deliveries, nerve damage, intrinsic sphincteric deficiency (ISD), body mass index (BMI), advancing age, hormonal changes, and smoking. The current treatment options mainly include conservative, pharmacological, and surgical treatments, and the efficacy is generally unsatisfactory. TERM has provided a variety of opportunities to restore the damaged sphincter function in patients with SUI (Table 2).

**TABLE 2:** TERM implants for PFD *in vitro* and *in vivo* (2015–2022).

Scaffold	Cell	Additive/treatment factor	Application	Main outcome
PGA	ADMSCs	5-Azacytidine	TE slings cultured *in vitro* and used in the SUI rat model	TE slings promoted collagen production, integrated better with the urethral sphincter, and rescued the urine controlling ability and the LPP of the rat model Wang et al. (2016)
PGA	ADMSCs		TE slings cultured *in vitro*	TE sling demonstrated matured form at 12 weeks, with gradually increased mechanical properties and collagen fibers and myoblast expression over time Wang et al. (2017)
PLACL/gelatin nanofiber meshes	SUSD2+ eMSC		Foreign body response of SUSD^2+^eMSCPLACL/gelatin meshes in the mice model	eMSCs impacted the degradation rate and tissue integration of PLACL/gelatin mesh and PLACL/gelatin nanofiber meshes enable entrapment of eMSCs for up to 6 weeks promoting substantial cellular infiltration of host anti-inflammatory macrophages Mukherjee et al. (2019)
PLA	ADSCs		Cell-impregnated scaffolds developed *in vitro* for the repair of SUI and POP	ADSCs attached well and increased in number and metabolic activity; ultimate tensile (UT) strength, UT strain, and YM of scaffolds increased; collagen I, collagen III, and elastin were produced at acceptable levels. Wang et al. (2018b)
PLA	ADMSCs and OF	Intermittent stress	*In vitro* TERM for POP and SUI	Both cells attached and proliferated well on PLA, increased biomechanical properties of scaffolds, and produced more elastin under restrained conditions. Under unstrained conditions, ADSCs on PLA produced more total collagen and a denser homogenous ECM than OF Roman et al. (2014)
PLA	ADMSCs	Estradiol	ADSCs seeded on estradiol-releasing scaffolds	PLA-estradiol scaffolds increased ECM production and stimulate angiogenesis (Mangir et al., 2019)
PLA	HDF	AA/A2P	PLA-AA/A2P scaffolds were co-cultured with fibroblasts	PLA-AA/A2P scaffolds increased hydrophilicity and strength and promoted collagen production of HDF Mangir et al. (2016)
PLA	hADMSCs		Construct scaffolds that mimic the 3D architecture of human fascia	PLA-aligned scaffolds showed increased bulk density, Young’s modulus, and UTS, promoted the production of collagen, and maintained the strength and stiffness without changes after 2 weeks of culture *in vitro*
PLA/PU	ADMSCs	Dynamic loading	Cell-impregnated sling *in vitro* for SUI	PLA/Z1 improved the interaction of the scaffolds with cells, reduction in material strength, and the ability of cells to penetrate the scaffolds Hillary et al. (2016)
PLGA	BMSCs	bFGF/mirRNA-29a-3p inhibition	BMSCs- mirRNA-29a-3p + PLGA- bFGF for PFD rats	MirRNA-29a-3p + PLGA-bFGF promoted elastin production of BMSCs, rescued the void volume, bladder void pressure, and LPP Jin et al. (2016b)
PLGA	BMSCs	bFGF + elastin-BMSCs	Elastin-BMSCs and PLGA-bFGF to the pelvis of PFD rats	PLGA-bFGF induced prolonged production of collagen and elastin from elastin–BMSCs, and PLGA-bFGF + elastin–BMSCs improved the urodynamic tests
MPEG-PLGA	MEF		MFF seeded on MPEG-PLGA scaffold for the rat abdominal wall defect model	Cells originating from the MFF influence the histological and biomechanical properties of the native tissue Jango et al. (2015). MPEG-PLGA + MFF explants showed higher stiffness and strength, with no sign of an inflammatory or foreign-body response
PCL	rMSC	bFGF/CTGF	PCL-CTGF-rMSC used on a rat model of PFD	PCL-CTGF-rMSC mesh showed increased biomechanical properties, collagen production, and without complications after 8 and 24 weeks Hansen et al. (2020)
PCL/PEG	ADSCs	Azithromycin	PCL/PEG–azithromycin mesh *in vitro*	PCL/PEG–azithromycin mesh showed anti-infectious properties and supported cell attachment and proliferation after pre-released for 14 days Ren et al. (2022)
PCL/PLGA	Fibroblasts		Effect of PCL/PLGA scaffolds on fibroblasts	Gentle cyclic straining of human fibroblasts on PCL/PLGA scaffolds enhances the regenerative potential
P (LLA-CL)-collagen 1 nanoyarn	Myoblasts from PSCs		Fabricate a novel nanoyarn for the treatment of SUI as a sling	P (LLA-CL)-collagen1 sling promoted the proliferation, infiltration, and production of collagen and elastin
PU	hADMSCs	17-β-estradiol	Developing scaffolds for POP and SUI	PU-17-β-estradiol scaffolds increased the ultimate tensile strength and promoted ECM production and angiogenic formation Shafaat et al. (2018)
PLCL	VE/SC		TE-based treatment for vaginal defects	VE/SC attached and maintained viability on scPLCL Sartoneva et al. (2018)
PCL/PLGA	VF		Effect of fiber diameter on scaffolds and cells *in vitro*	Fiber diameter affects cell behavior, ECM deposition, and the mechanical properties of the matrices but did not affect the ultimate tensile strength Vashaghian et al. (2016)
PTLG	ADSCs		Cell-impregnated scaffolds for repair of SUI and POP	ADSCs attached well and increased in number and metabolic activity; ultimate tensile (UT) strength, UT strain, and YM of scaffolds increased; collagen I, collagen III, and elastin were produced at acceptable levels. Wang et al. (2018b)

The suburethral sling implantation is the mainstay of surgical treatment for SUI. However, these approaches only supply passive support for the urethral lumen without restoring the damaged sphincter function. Also, tissue rejection, urethral erosion, and infection are the major problems and headaches of the currently used slings. Ying Wang tried to explore the possibility of generating a sling complex *in vitro* by seeding ADSCs onto PGA under constant strain; the results showed that the ADSC-PGA construct appeared as a sling-like structure with a cord-like shape during the first 4 weeks of *in vitro* culture, and the ADSC-PGA sling exhibited to be relatively thicker, smoother, much thinner, and mature at 12 weeks and had gradually increased stress/strain curves, max load, and Young’s modulus values over time. The collagen fibers and myoblasts’ expression increased in a time-dependent manner (Wang et al., 2017). A tissue-engineered sling was constructed by seeding GFP-transfected ADSCs on PGA fibers, and with the induction of 5-azacytidine for 4 weeks, the sling was then implanted in the SUI rat model established by the vaginal balloon dilatation method and bilateral ovariectomy. LPP increased significantly and reached nearly close to baseline normal levels, 2 months after implantation. ADSCs promote more collagen matrix formation and are integrated better with the urethral sphincter (Wang et al., 2016). To find materials with more appropriate mechanical properties for a sling, which can also support cell integration, scaffolds of different materials were compared; the results showed that PU Z1 and PU Z3 coped well with dynamic strain and maintained their elasticity after 7 days of sustained cyclical distention (Hillary et al., 2016). PLA is superior at supporting cellular interactions and new matrix production, but the mechanical properties changed after only 7 days of dynamic strain. PU/PLA was weaker and stiffer than PU or PPL but significantly improved the interaction of the scaffolds with cells and promoted the total collagen expression of hADSCs cultured on the scaffolds, suggesting that a combination of materials could be more suitable for successful implantation and longer survival for the management of SUI. Zhang et al. (2016) fabricated a novel electrospun nanoyarn with higher porosity, larger pore size, and aligned fibers/yarns than the nanofiber scaffold. A tissue-engineered sling was formed by seeding myoblasts on nanoyarn, and the myoblasts proliferated greatly on the nanoyarn scaffold and infiltrated deeply into it after 7 days; nanoyarn myoblasts produced more type 1 and 3 collagen and elastin and improved muscular tissue development, suggesting myoblast–nanoyarn sling could be a promising tissue-engineered sling for SUI.

Bulking agents are another common treatment for SUI, which allows minimally invasive treatment and low medical expenses. However, the migration and/or degradation of the injected bulking agents shortened the lasting of long-term effectiveness in the therapeutic period. An alternative approach based on cells/bioactive molecules and biodegradable biomaterials can induce the regeneration of target tissues and improve the sphincter function. Oh et al. (2015) developed an injectable bioactive bulking agent for the SUI rat model by loading dual growth factor (NGF and bFGF) *in situ* hydrogel/PCL bead mixture, and the results showed that the PCL beads were located stably at the applied site without migration. The sequential release of the growth factors from the bulking agent promoted the regeneration of damaged nerves and smooth muscles and thus enhanced biological function around the urethra. Jin et al. (2016a) formulated the bFGF-loaded PLGA nanoparticle (bFGF-loaded PLGA NPs) system to achieve the sustained release of bFGF and employed this system in elastin–over-expressing BMSC culture *in vitro* and in the rat vaginal distention translational model *in vivo*. The results showed that bFGF-loaded PLGA NPs could markedly stimulate the differentiation of elastin–over-expressing BMSCs to fibroblasts and then produce more collagen and elastin *in vitro* and *in vivo*. Also, the combination using of a bFGF-loaded PLGA NP system and elastin–over-expressing BMSCs in the rat model could alleviate the PFD symptoms, reverse the decreased void volume and bladder void pressure, and rescue the decreased peak bladder pressure. In another study, Jin et al. (2016b) demonstrated that inhibition of miR-29a-3p in BMSCs along with bFGF-PLGA-NP injection can markedly increase the expression and secretion of elastin, which consequently largely improved the urodynamic parameters and urodynamics and promoted the therapeutic effect of BMSCs on PFD rats.

### Tissue-engineered repair material for pelvic organ prolapse

POP is defined as the descent of one or more of the pelvic organs (vaginal wall, bladder, uterus, and vaginal apex) from their natural positions in the pelvis because of the weakening of the pelvic floor and the organ support structure (Iglesia and Smithling 2017). POP mainly results from pregnancy/vaginal birth, which is due to the strain, overstretching, and tearing of the pelvic floor ligaments, muscles, and connective tissues (Wallace et al., 2019). Surgery treatment with synthetic meshes is the main method for POP therapy, but the surgical outcomes do not meet patients’ needs. Many studies demonstrated that TERM consisting of cells/drugs/growth factors/biodegradable scaffolds could improve the therapeutic effect (Table 2).

Jango et al. (2015) employed MPEG-PLGA (methoxypolyethyleneglycol polylactic-co-glycolic acid) scaffold seeded with autologous muscle fiber fragments (MFFs) to treat a rat abdominal wall defect model; the results demonstrated that MPEG-PLGA scaffold is a safe carrier for MFFs, and MFFs affect the regenerative repair process, but the MPEG-PLGA+MFF group showed a trend toward higher stiffness in the physiologically more important low stiffness zone, which is not ideal from a clinical point of view. An estradiol-releasing electrospun PLA mesh containing different concentrations of estradiol was designed, and the results showed that ADMSCs cultured on estradiol-releasing PLA meshes produced more ECM and formed more new blood vessels and outgrew the pro-angiogenic cells (Mangir et al., 2019). Meanwhile, the estradiol-releasing PLA mesh with ADMSCs was more elastic and stronger than control PLA meshes. PU scaffolds with 17-β-estradiol significantly increased the ultimate tensile strength of scaffolds, and hADMSCs on estradiol-releasing PU scaffolds showed more ECM production, higher angiogenic potential, and good cellular infiltration and tissue integration (Shafaat et al., 2018). Ascorbic acid (AA) and ascorbate-2-phosphate (A2P) containing PLA scaffolds were significantly more hydrophilic and stronger than PLA scaffolds and had approximately two times higher UTS, strain, and YM values than pure PLA. Human dermal fibroblasts seeded on AA or A2P containing PLA scaffolds produced more collagen (Mangir et al., 2016). Four different PLA scaffolds with various degrees of fiber alignment were constructed to mimic the three-dimensional architecture of human fascia, and the results showed that the bulk density, Young’s modulus, and UTS were significantly increased from PLA-random to PLA-aligned scaffolds, and ADSCs grew well on scaffolds with aligned fibers, produced the largest amount of total collagen, and maintained the strength and stiffness without changes after 2 weeks of culture *in vitro* (Roman et al., 2016). The scPLCL is a potential scaffold material for vaginal tissue engineering, vaginal epithelial (VE), and stromal cells (SCs) that attached and maintained viability on scPLCL (Sartoneva et al., 2018). The comparison of PLGA/PCL films with different fiber diameters suggested that the fiber diameter affects cell behavior, mechanical properties, and cellular infiltration ECM deposition (Vashaghian et al., 2016).

## Conclusion

The clinical experience and studies *in vitro* or *in vivo* suggest that tissue engineering technology can provide successful outcomes when used in the surgical management of pelvic floor disorders. These data provide valuable evidence for clinical application in the urogynecological setting. However, different cells, scaffolds, or cell-combined scaffolds have their own advantages and disadvantages, which can produce different therapeutic effects on PFD. The question is which is most desirable? Therefore, more studies with longer follow-up are needed to select the most effective and safe cells and materials for PFD repair.
